# Wearing a mask—For yourself or for others? Behavioral correlates of mask wearing among COVID-19 frontline workers

**DOI:** 10.1371/journal.pone.0253621

**Published:** 2021-07-19

**Authors:** Ankush Asri, Viola Asri, Baiba Renerte, Franziska Föllmi-Heusi, Joerg D. Leuppi, Juergen Muser, Reto Nüesch, Dominik Schuler, Urs Fischbacher

**Affiliations:** 1 University of Konstanz, Konstanz, Germany; 2 Thurgau Institute of Economics, Kreuzlingen, Switzerland; 3 Spital Schwyz, Schwyz, Switzerland; 4 Swiss Institute for International Economics and Applied Economic Research, University of St.Gallen, St.Gallen, Switzerland; 5 Kantonsspital Baselland, Liestal, Switzerland; 6 University of Basel, Basel, Switzerland; Universidad Loyola Andalucia Cordoba, SPAIN

## Abstract

Human behavior can have effects on oneself and externalities on others. Mask wearing is such a behavior in the current pandemic. What motivates people to wear face masks in public when mask wearing is voluntary or not enforced? Which benefits should the policy makers rather emphasize in information campaigns—the reduced chances of getting the SARS-CoV-2 virus (benefits for oneself) or the reduced chances of transmitting the virus (benefits for others in the society)? In this paper, we link measured risk preferences and other-regarding preferences to mask wearing habits among 840 surveyed employees of two large Swiss hospitals. We find that the leading mask-wearing motivations change with age: While for older people, mask wearing habits are best explained by their self-regarding risk preferences, younger people are also motivated by other-regarding concerns. Our results are robust to different specifications including linear probability models, probit models and Lasso covariate selection models. Our findings thus allow drawing policy implications for effectively communicating public-health recommendations to frontline workers during the COVID-19 pandemic.

## Introduction

The World Health Organization (WHO) declared the novel SARS-CoV-2 coronavirus disease 2019 (COVID-19) outbreak a pandemic on the 11^th^ of March 2020 [1], and face masks swiftly became one of the most visible symbols of the pandemic [2, 3]. The emerging consensus is that mask wearing reduces the chances of both catching and spreading the virus and thus protects both the wearer herself and other people around her [4–7].

Yet, human behavior is central to how well masks work [8–12]. The studies on efficacy of mask wearing rely on the assumptions that people wear masks—and wear them correctly [13]. Meanwhile, COVID-19 provides different intrinsic motivations for mask wearing for different age groups, as older people face a considerably higher mortality risk from disease complications than younger people [14]. Accordingly, an intergenerational conflict in motivations for complying with the pandemic restrictions, including mask wearing, is likely to occur. We thus hypothesize that tailored strategies for communicating public health recommendations regarding mask wearing to the older and younger age groups are needed.

At the beginning of the pandemic, medical experts lacked evidence on how the virus spreads, leading to inconsistencies in public health recommendations from health organizations and political leaders [15–17]. For example, the WHO and the Swiss Federal Office of Public Health (FOPH) initially refrained from endorsing mask wearing, partly due to the lacking evidence and partly due to worries about depleting supplies for frontline workers. In March 2020, the WHO even stated that people who “are not ill or looking after someone who is ill” would be “wasting a mask” if they wore one [18]. The Swiss FOPH echoed this position stating that “healthy people should not wear hygiene masks (surgical masks) in public” [19]. This opinion changed in late April 2020, when the FOPH issued a recommendation to wear masks when physical distancing is not possible, and the WHO issued a similar recommendation in June 2020 [20, 21].

Given these inconsistencies in public messages and intrinsic motivations, it is now more important than ever to develop an effective strategy for communicating public health recommendations regarding the importance of mask wearing. It is particularly important to understand how to motivate people of different ages to wear masks in public when mask wearing is voluntary. People could be motivated to wear masks either to benefit themselves by not catching the virus (risk preferences) or to benefit others by not transmitting it (other-regarding preferences). It is not yet established, however, which motivation dominates in which context. Given that masks can save lives in different ways, which benefits to rather stress in public messages to specific target groups—the reduced chances of catching or transmitting the virus?

In this study, we link measured risk preferences and other-regarding preferences to voluntary mask wearing habits outside work among COVID-19 frontline workers in two Swiss hospitals. Switzerland is well suited for our study due to the unique combination of two aspects—the varying rate of COVID-19 infections and the stringency level of government response. In terms of the infection rates, Switzerland was temporarily one of the most affected countries in the world (per capita on average) during the first COVID-19 wave of 2020 [22]. Due to Switzerland’s heavy reliance on cross-border workers, the central and less populous regions of Switzerland were significantly less affected than the border regions throughout the first wave [22]. Nevertheless, according to the Government Response Stringency Index [23], the Swiss FOPH has remained somewhat more lenient in terms of the anti-COVID-19 restrictions than its counterparts in the neighboring countries. For example, mask wearing was mandatory for general public only in the public transport and only starting July 2020. Although some regions of Switzerland either lifted or introduced some other measures to limit the spread of COVID-19 during the period of interest for this study, no significant policy changes took place in our regions of interest; see the Methods section for a detailed timeline.

Healthcare workers, as an exemplary population, are particularly suited for our inquiry for a number of reasons. First, all employees of Swiss hospitals were required to wear masks at work, such that they were used to wearing them on a daily basis and had easy access to mask supplies. Second, acting at the frontline of the COVID-19 crisis, they were comparatively well informed about the benefits of mask wearing and risks that follow from not wearing a mask. Third, potentially having been closer to people who have been infected with the SARS-CoV-2 virus, the hospital employees might have been exceptionally motivated to wear masks in public even if mask wearing rules were voluntary.

And yet, quite curiously, there is considerable heterogeneity in mask wearing habits even within this population. Previous studies have shed some light on the potential motivations and characteristics that could explain this variation. On the one hand, people who wear masks tend to be more empathic [24], more conform to societal norms [25, 26], more reliant on reasoning rather than emotions [27], and more willing to re-establish a sense of control, as people reportedly feel relief from anxiety when wearing masks [25]. On the other hand, people who avoid endorsing or complying with prevention measures, including mask wearing, tend to be more risk-taking, callous and dishonest [28, 29] and even exhibit more hypermasculinity [30] and so-called dark traits (e.g., psychopathy) [31, 32]. While studies often focus on these motivations separately, it is not yet clear which motivations for mask wearing behavior dominates when comparing them for different age groups.

On the first glance, it might seem that the benefits for oneself comprise a stronger argument for compliance. However, previous studies that compare self-regarding and other-regarding motivations for health behavior provide mixed results [33–35]. Indeed, humans are social beings and have the willingness, need and ability to cooperate with others, even beyond own kin [36–39]. It is for this reason that people are reportedly motivated to restrict their everyday lives to protect others who belong to a COVID-19 risk group [9], and it is for this reason that appealing to people’s other-regarding motivations in the context of mask wearing might be the “stronger public health rationale” [40].

To address this question, we conducted a survey of Swiss healthcare workers right after the first COVID-19 wave of 2020 to examine the various motivations behind voluntary mask wearing. We focused on two hospitals of regional importance in two differently affected regions: one larger hospital with ca. 3500 employees in the northwest part of Switzerland, which was an area with moderately high COVID-19 incidence per capita during the first COVID-19 wave (henceforth “more-affected region”) and one smaller hospital with ca. 500 employees in the central part of Switzerland, where COVID-19 incidence per capita was relatively low (henceforth “less-affected region”); please refer to the Methods section for further details on COVID-19 prevalence in each region and the survey design. While mask wearing was compulsory within both hospitals throughout the whole data collection period in mid-2020, it was recommended but not compulsory outside of the hospitals (e.g., in supermarkets or other crowded places) in the respective regions over this time period.

Accordingly, as we will elaborate in the Results section, voluntary mask wearing habits had developed differently in the two regions, in that mask wearing is higher on average in the more-affected region compared to the less-affected region (in line with the literature on exposure [41], which suggests, e.g., that outbreaks lead to increased vaccination rates). We can thus examine how mask wearing motivations—for oneself or for others—differ given the two different equilibria in (i) the more-affected region where the social norm leans towards wearing masks and (ii) the less-affected region where the social norm leans towards not wearing masks. Our main variables of interest include a risk preference measure [42] and an other-regarding altruism measure [43], and we hypothesize that both higher risk aversion and higher altruism lead to more mask wearing, although their importance differs. Our main comparisons of interest include between-region and, given the specifics of COVID-19 risk, between-age-group comparisons.

## Materials and methods

**Fig 1:**
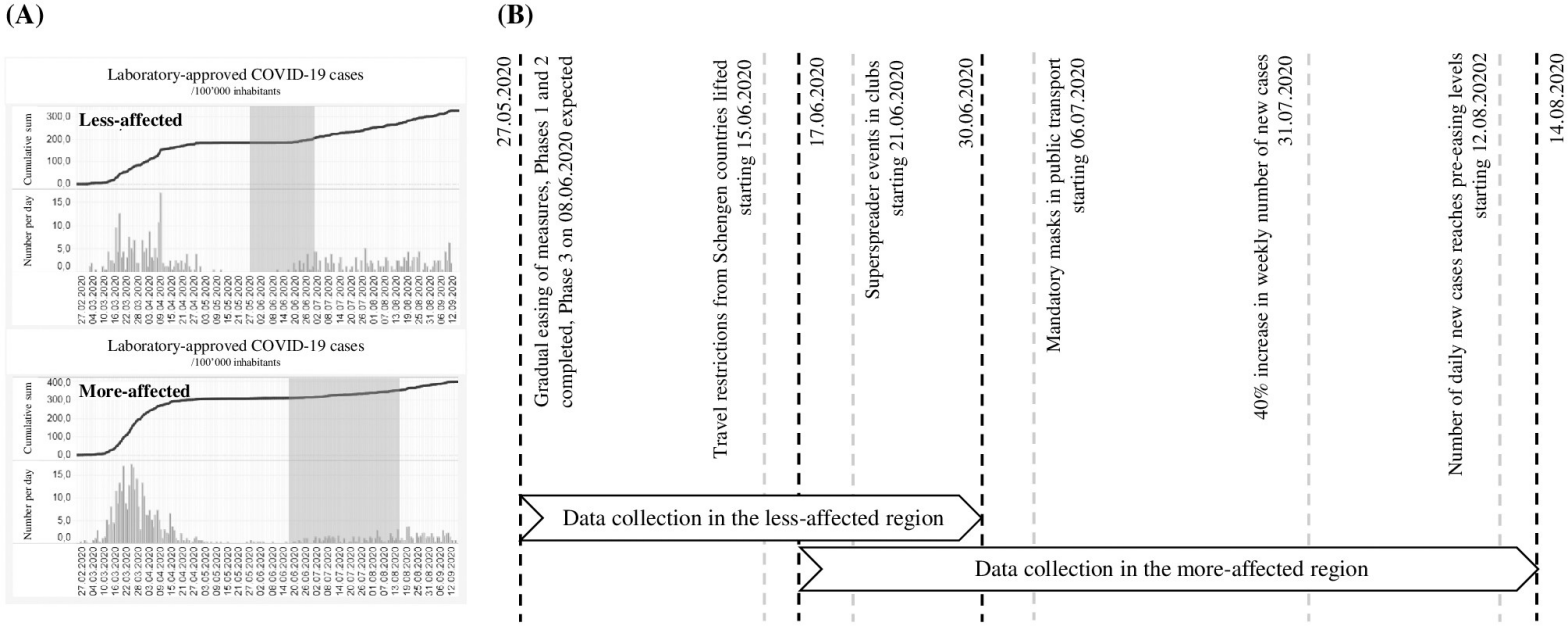
Timeline. (A) Development of COVID-19 in the less-affected and more-affected region relative to data collection periods, according to FOPH (B) Timeline of the study and COVID-19 policy milestones and debates in Switzerland.

We carried out an online survey of employees from two hospitals in Switzerland—Spital Schwyz and Kantonsspital Baselland—to analyze behavioral correlates of compliance with COVID-19 preventive measures and prevalence of COVID-19 infections. Informed consent was obtained from all participants. Ethical approval for this study was waived by the Ethics Committee Northwest and Central Switzerland. Hospital employees were invited by mail to their home address to participate in the online survey. Overall, 840 hospital employees participated in the survey: 540 from the more-affected region and 300 from the less-affected region. The survey took place from the end of May 2020 to the end of June 2020 in the hospital in the less-affected region and from mid-June to mid-August 2020 in the hospital in the more-affected region; see the timeline in Fig 1 below. The online survey was in German, given that the working language in both hospitals is German and the hospital management confirmed that all hospital employees are sufficiently fluent in German.

In the online survey, the hospital employees reported their voluntary mask wearing habits as well as answered general questions about the two competing preferences potentially motivating mask wearing: risk aversion and altruism. We analyze the data from both hospitals separately, considering the two hospitals as two different clusters. We use multivariate regression analysis to examine the motivations predicting mask wearing. The dependent variable is *mask wearing*. Survey respondents were asked “Do you currently wear a mask when you go to supermarkets or other crowded places?” and could respond “Yes”, “Sometimes” or “No”. For the main analysis, we code the binary dependent variable as 1 if the respondent says “Yes” or “Sometimes” and as 0 if the respondent says “No”. In addition, we perform robustness checks with different specifications and report these in the S1 Appendix. As the dependent variable is binary, we use linear probability models with heteroskedasticity-robust standard errors [44]. The unit of observation is the hospital employee *i*. The independent variables of interest are self-reported risk preference and self-reported altruism as measured in the online survey.

We start the empirical analysis by presenting how the percentage of people reporting to wear a mask varies by our variables of interest—risk preference and altruism—as well as the hypothesized moderating variable age. We then split the sample into younger and older employees to examine the mask-wearing rates among the younger and older employees for risk averse, risk seeking, altruistic and not-altruistic individuals.

We then proceed to examine the two competing motivations for mask wearing by regressing mask wearing as a binary variable on risk aversion and pure altruism in a linear probability model as follows:

$$\begin{array}{c} {\text{Mask}\;\text{wearing}}_{i}=\beta_{0}+\beta_{1}\text{Risk}\;{\text{aversion}}_{i}+\beta_{2}{\text{Altruism}}_{i}+X_{i}^{\prime }\gamma +\epsilon_{i} \end{array}$$ (1)

The parameters of interest are *β*_1_ and *β*_2_ denoting the correlation of risk aversion and pure altruism with mask wearing. $X_{i}^{\prime }$ denotes the vector of covariates. Individual covariates include occupational group, age, education, native and gender. Situational covariates include international travel, using public transport, having been in contact with COVID-19 infected person at work or outside work, belonging to a COVID-19 risk group and having a household member who belongs to a COVID-19 risk group.

Subsequently, we examine whether and to what extent the motivation varies with the age of the respondent. We split the sample into younger and older hospital employees at the median and include interaction terms between the independent variable of interest and belonging to the younger or older in the regression, using again a linear probability model:

$$\begin{array}{c} {\text{Mask}\;\text{wearing}}_{i}=\beta_{0}+\beta_{1}\text{Risk}\;{\text{aversion}}_{i}\cdot {\text{Older}}_{i}+\beta_{2}{\text{Altruism}}_{i}\cdot {\text{Older}}_{i}\\ +\beta_{3}\text{Risk}\;{\text{aversion}}_{i}+\beta_{4}{\text{Altruism}}_{i}+X_{i}^{\prime }\gamma +\epsilon_{i} \end{array}$$ (2)

From this regression, we obtain parameters *β*_3_ and *β*_4_ indicating the correlation of risk aversion and altruism with mask wearing for younger employees. The linear combinations *β*_1_ + *β*_3_ and *β*_2_ + *β*_4_ indicate the association between mask wearing and risk aversion for older hospital employees.

In line with the econometric literature on regressions for binary outcome variables, the linear probability model is the preferred specification [44]. We additionally provide the results from probit models with marginal effects and from a data-driven approach for the selection of covariates using Lasso regressions [45, 46] in the S1 Appendix.

Below, we provide a description of the independent variables of interest. The measures for self-reported risk preference and altruism have been extensively validated in previous studies [43, 47, 48].

### Self-reported general risk aversion

We use the well-established survey question from the German socio-economic panel: “How do you see yourself? Are you in general a person willing to take risks or as a person who tries to avoid risks?”. Respondents answer on a Likert-scale from 0 to 10 [42, 47, 48].

### Self-reported altruism

We use the well-established survey question from the Global Preferences Survey. Respondents answer on a Likert-scale from 0 to 10 indicating how well a statement describes them as a person. For pure altruism, the statement is “I am willing to donate to good causes without expecting anything in return.” [43].

## Results

We examine what motivates frontline workers to wear a face mask in the public outside their workplace. We use the data of both hospitals, one located in the more-affected region (N = 540) and the other one located in the less-affected region (N = 300), but analyze the data from both locations separately considering both hospitals as two different clusters; please see Table 1 in S1 Appendix for the survey questions and Table 2 in S1 Appendix for summary statistics. We find that the relevance of the two competing motivations for voluntary mask wearing varies between the regions. Moreover, the importance of these motivations varies with age: While older people are motivated by risk aversion, younger people are also motivated by other-regarding concerns, and this comparison is more pronounced in the more-affected region.

**Fig 2:**
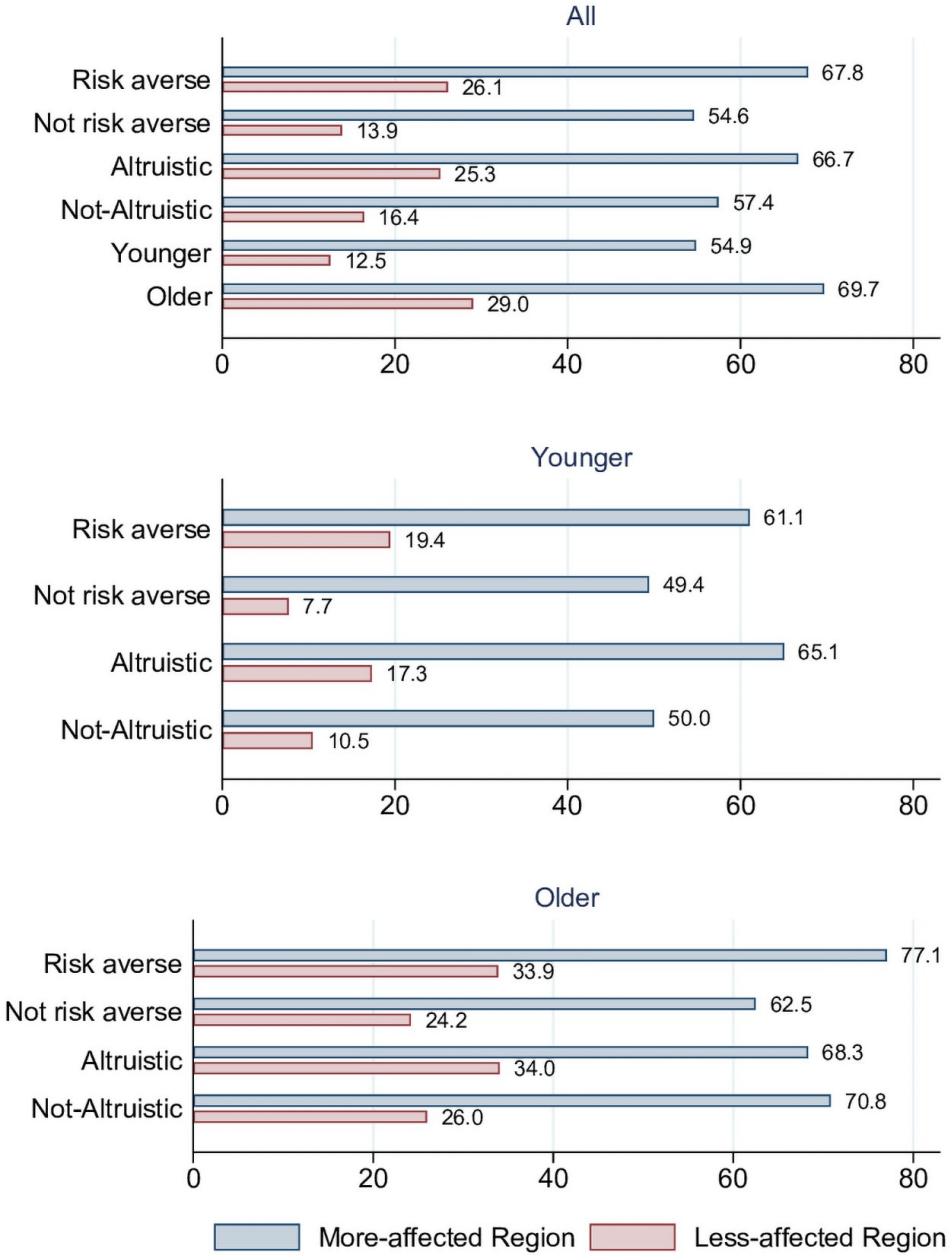
Descriptive statistics on mask wearing in percent. Risk aversion and altruism are binary variables standardized at median. “Younger” refers to up to 44 years of age and “Older” refers to 45 years of age and older.

First, we examine how the percentage of respondents reporting to wear a mask varies by the two variables of interest—risk preference and altruism—as well as by age. Overall, in the more-affected region, 60.9% of the survey respondents wear a mask. In the less-affected region, 19.3% wear a mask (p-value<0.001). As shown in Fig 2, mask wearing varies by risk preference and altruism. Mask wearing is more common among risk-averse individuals than among risk-seeking individuals: 67.8% compared to 54.6% in the more-affected region (p-value = 0.00 2) and 26.1% compared to 13.9% in the less-affected region (p-value = 0.007). Similarly, altruistic individuals report wearing a mask more often than not-altruistic individuals: 66.7% compared to 57.4% in the more-affected region (p-value = 0.033) and 25.3% compared to 16.4% in the less-affected region (p-value = 0.068). As the motivations for mask wearing may vary by age, we further note that in general younger employees report wearing a mask less often than older employees: 54.9% compared to 69.7% in the more-affected region (p-value<0.001) and 12.5% compared to 29.0% in the less-affected region (p-value<0.001).

Splitting the sample by age now in each of the two hospitals shows descriptively that, for younger individuals, mask wearing varies both by risk-aversion and altruism, with the motivation stemming from other-regarding preferences appearing more pronounced in more-affected region than in the less affected region. In the more affected region, 61.1% of the young risk-averse individuals report wearing a mask compared to 49.4% of the young risk-seeking individuals (p-value = 0.037). A similar pattern is visible in the less-affected region with 19.4% of the young risk-averse individuals reporting to wear a mask and 7.7% of the young risk-seeking individuals (p-value = 0.020). Further, 65.1% of the young altruistic individuals report wearing a mask compared to 50.0% of the young non-altruistic people report wearing a mask in the more affected region (p-value = 0.012) and 17.3% compared to 10.5% in the less affected region (p-value = 0.214).

For older employees, mask wearing varies only by risk aversion and not by altruism, and the self-protecting motivation appears to be more pronounced in the more-affected region. Among the risk-averse older individuals, 77.1% wear a mask compared to 62.5% of the risk-seeking older individuals (p-value = 0.018) in the more affected region. The difference is insignificant in the less-affected region (p-value = 0.239). For older employees, mask wearing does not seem to vary with altruism (p-value = 0.687 in the more-affected region and p-value = 0.341 in the less-affected region).

**Fig 3:**
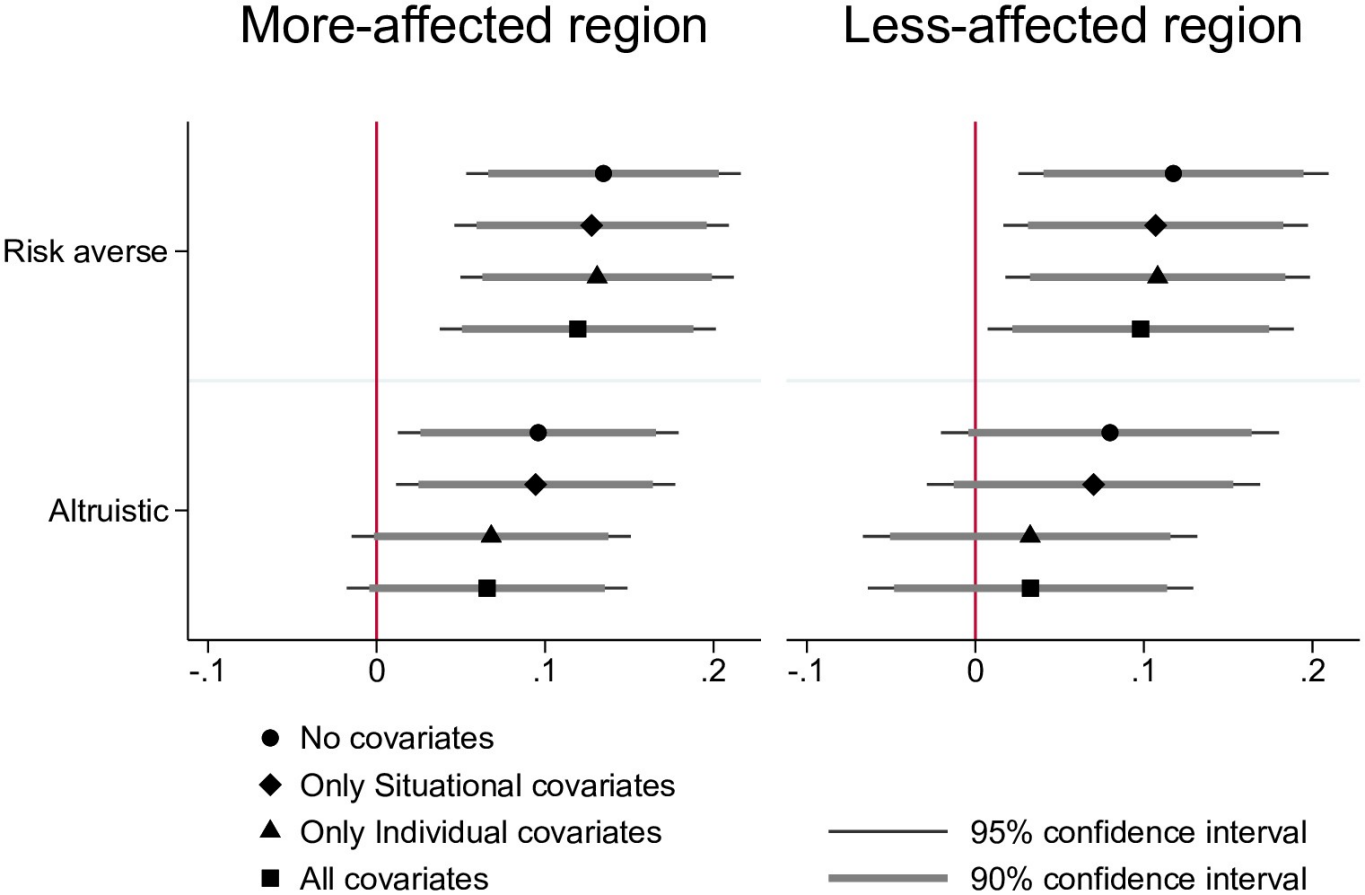
Motivations behind mask wearing. In this figure, we visualize the results from regressing mask wearing on the variables of interest risk aversion and altruism. The x-axis shows the estimate for the coefficient on risk aversion and the estimate for the coefficient on altruism first without covariates, then including only situational covariates, then including only individual covariates, and finally including both. The thin line represents the 95% confidence interval and the thick line represents the 90% confidence interval. Risk aversion and altruism are binary variables standardized at median. Situational covariates include having had COVID-19 symptoms, household size, using public transport, having had contact to a COVID-19 infected person at work, having had contact to a COVID-19 infected person outside work, belonging to a COVID-19 risk group, living with a household member who belongs to a COVID-19 risk group, having traveled internationally for at least 2 days since 1st of February 2020. Individual covariates include being a health worker (doctor or nurse), age group, education group (low, medium or high level of education), being native and gender.

We model the two potentially competing motivations—risk aversion and altruism—by regressing mask wearing as a binary variable on risk aversion and altruism standardized at median in an ordinary least squares (OLS) linear regression model; please see Tables 3 to 5 in S1 Appendix for further details. We further account for individual and situational covariates as predictors of mask wearing. As depicted in Fig 3, both in the more-affected region and in the less-affected region, risk aversion and altruism are both positively correlated with mask wearing. In terms of significance and accounting for other predictors, risk aversion predicts mask wearing more strongly than altruism, suggesting—at least at the first sight—that risk preferences are the main drivers of mask wearing in the context of the COVID-19 pandemic. After controlling for individual and situational covariates, being risk-averse is associated with an increase in the likelihood of wearing a mask by 13.0 percentage points (p-value = 0.008) in the more-affected region and by 8.4 percentage points (p-value = 0.063) in the less affected region. In the more-affected region, being altruistic is associated with an increase in the likelihood of mask wearing by 9.6 percentage points increase (p-value = 0.023) but it is a not significant predictor once we account for individual covariates, neither when we focus on the frontline workers in the less-affected region. We summarize this initial finding as Result 1.

**Result 1: People who are more risk-averse are more likely to wear a mask**.

However, given the specifics of the known COVID-19 risks, Result 1 on the importance of risk aversion as the primary motivation behind mask wearing may neglect important differences between age groups. While avoiding the risk of getting infected may be the central motivation for older employees who tend to be more at risk of experiencing serious symptoms, younger employees might be less concerned about catching the disease themselves and instead more concerned about older people suffering from serious symptoms.

**Fig 4:**
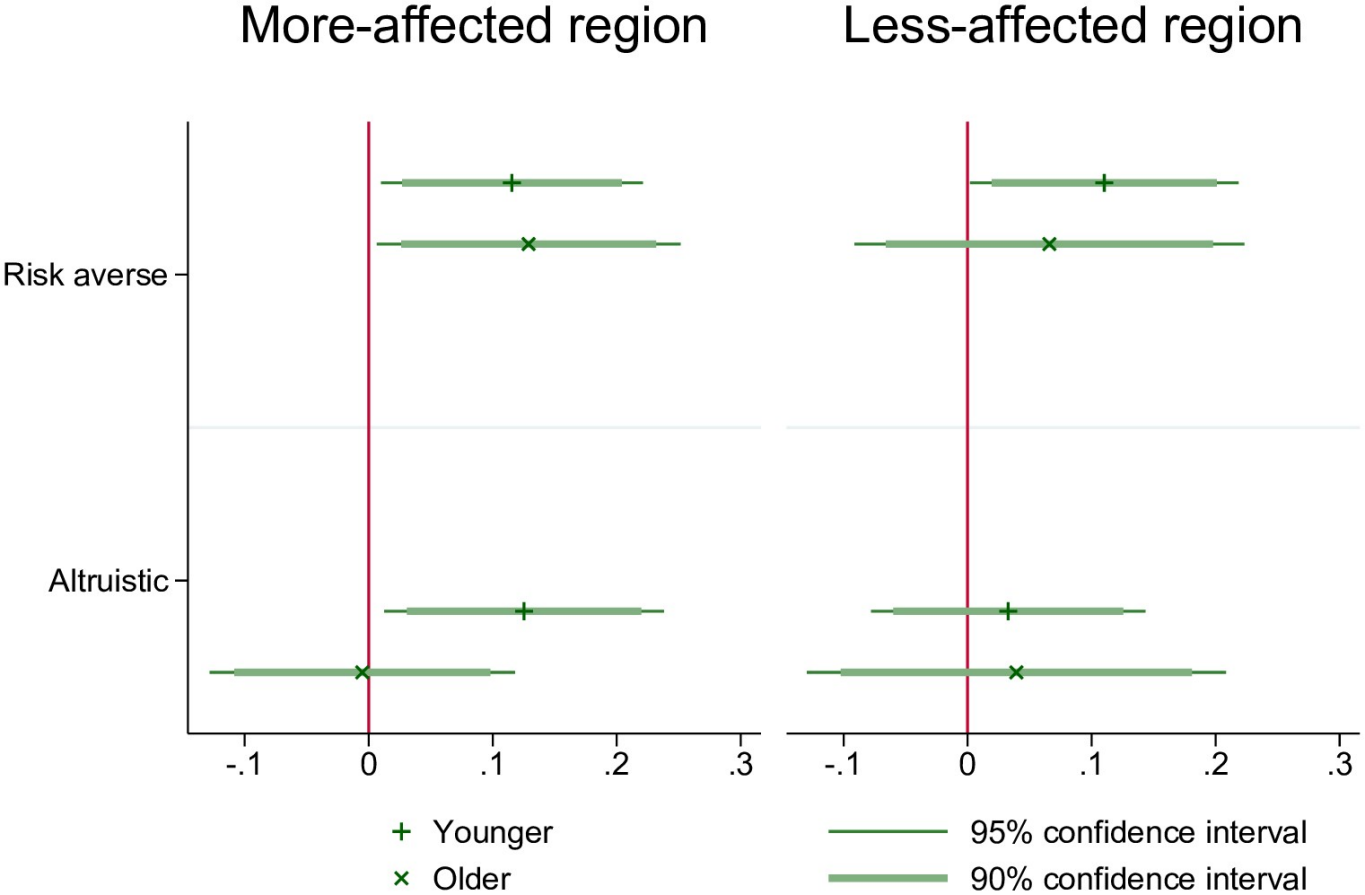
Motivations behind mask wearing, split by age. The presented estimates are from regressions with interaction terms between the variable of interest and being younger, controlling for all situational and individual covariates. Situational covariates include having had COVID-19 symptoms, household size, using public transport, having had contact to a COVID-19 infected person at work, having had contact to a COVID-19 infected person outside work, belonging to a COVID-19 risk group, living with a household member who belongs to a COVID-19 risk group, having traveled internationally for at least 2 days since 1st of February 2020. Individual covariates include being a health worker (doctor or nurse), age group, education group (low, medium or high level of education), being native and gender. See also the comments on Fig 3.

Given these differences, we use interaction terms of risk aversion and altruism with age to examine whether the two motivations, risk aversion and altruism, vary in their relevance for younger and older hospital employees. As shown in Fig 4, in the more-affected region, risk aversion is associated with a higher likelihood to wear a mask for both younger and older hospital employees. Younger employees who are risk-averse are 12.5 percentage points (p-value = 0.043) more likely to wear a mask than their risk-seeing counterparts. Older employees who are risk-averse are 14.1 percentage points (p-value = 0.060) more likely to wear a mask than their risk-seeking counterparts. In the less-affected region, risk aversion is only associated with higher likelihood of wearing a mask for the younger hospital employees. Younger risk-averse employees are 9 percentage points (p-value = 0.068) more likely to wear a mask than their risk-seeking counterparts. Finally, we observe that other-regarding preferences, captured by altruism, influence mask wearing for the younger people in the more-affected region but not for younger people in the less-affected region. In the more-affected region, younger employees who are altruistic are 13 percentage points (p-value = 0.021) more likely to wear a mask than their non-altruistic counterparts. In other words, while older hospital employees tend to wear masks to protect themselves in the more-affected region, younger hospital employees are also motivated by altruistic motivations, but only in the more-affected region.

**Result 2: In the more-affected region, younger employees who are more altruistic are more likely to wear masks. They are motivated not only by risk aversion but also by other-regarding preferences (altruism)**.

Potentially, this result could be related to the two different equilibria prevailing in the more-affected region and in the less-affected region. While in the more-affected region, the social norm is to wear a mask at crowded places, in the less-affected region the social norm is not to wear a mask. Hence, other-regarding preferences are more likely to motivate people in the region where mask wearing is the norm compared to the region where mask wearing was not (yet) the norm.

Our results on how the motivations for mask wearing may change with age are robust to using continuous independent variables for risk aversion and altruism, considering the dependent variable for mask wearing as a three-level categorical variable, changing the functional form of the regression using probit regressions instead of linear probability models, and using a data driven approach in selecting the covariates using Lasso regressions [45, 46] Please see Tables 6 to 16 in S1 Appendix for further details.

## Conclusion

We have demonstrated the importance of ambidextrous strategies for communicating public health recommendations during the COVID-19 pandemic. We highlight different motivations for mask wearing for different subgroups of the frontline healthcare-worker population. Namely, both risk preferences and social preferences appear to play a role for the younger people, while predominantly risk preferences are related to mask wearing habits for middle-aged and older people. In our study, we have focused on a particularly important group of the population—hospital employees—for whom mask wearing is likely less affected by practical barriers, such as lack of access to mask supplies, lack of knowledge about the efficacy of masks or lack of experience with wearing masks. Accordingly, we argue that the effect of the different preferences in our study is less confounded by such additional factors.

Importantly, our analysis reveals that age is the only demographic measure related to significant differences in mask wearing habits—other measures like gender, education or occupation showed relatively minor differences. Our results thus align with the statistics on disease complications and mortality, by which older people are more likely to belong to the COVID-19 risk group. Our median cutoff for the younger and older subgroups also happens to coincide with the age group with 1% or higher COVID-19 mortality rate [14].

Our results hold also after we control for whether our respondents co-reside with a COVID-19 risk group member. We can thus conclude that the other-regarding preferences that we capture using our survey design apply to “others” beyond one’s own kin [49]. One useful extension of our study could thus include an investigation into further social motivations for mask wearing, including image concerns [50, 51] and coordination [52, 53].

We have measured general preferences, instead of focusing on exact messages [34], and contrast risk-related and altruism-related motivations as such. Accordingly, our research could also bring insights to situations that span beyond voluntary mask wearing settings. Previous research on the use of masks in non-healthcare settings relates to influenza-like illnesses and focus predominantly on the protection of the mask wearers themselves [54]. In the meantime, some studies argue that mass masking has not been more widely accepted because it provides moderate benefits to individuals and large benefits to whole populations [55]. Other examples of this paradox include, for example, vaccinations, which provide protection to oneself but require a high compliance rate to achieve herd immunity for others [56]. Aside from healthcare matters, also safe driving can be compared to mask wearing in this regard, in that other road users and pedestrians benefit from safe driving just as the driver herself does [40]. In general, our findings add yet another example for using social and behavioral science to support the COVID-19 pandemic response [57]. Yet, another useful extension of our study design could include other target groups of the population beyond frontline workers in the healthcare sector.

## Supporting information

S1 Appendix The supplementary materials provided separately include all survey questions, summary statistics and regression tables for the analyses in the paper. (PDF)

## References

[1] Mahase E. COVID-19: WHO declares pandemic because of “alarming levels” of spread, severity, and inaction. BMJ (Clinical research ed). 2020. 32165426 10.1136/bmj.m1036

[2] Sunstein CR. The meaning of masks. Journal of Behavioral Economics for Policy (COVID-19 Special Issue). 2020;4:5–8.

[3] Goh Y, Tan BYQ, Bhartendu C, Ong JJY, Sharma VK. The face mask: How a real protection becomes a psychological symbol during COVID-19?; 2020. 10.1016/j.bbi.2020.05.060 PMC7833005 32526447

[4] Liang M, Gao L, Cheng C, Zhou Q, Uy JP, Heiner K, et al. Efficacy of face mask in preventing respiratory virus transmission: A systematic review and meta-analysis. Travel Medicine and Infectious Disease. 2020;36:101751. doi: 10.1016/j.tmaid.2020.101751 32473312 PMC7253999

[5] Prather KA, Wang CC, Schooley RT. Reducing transmission of SARS-CoV-2. Science. 2020. doi: 10.1126/science.abc6197 32461212

[6] Hendrix MJ, Walde C, Findley K, Trotman R. Absence of apparent transmission of SARS-CoV-2 from two stylists after exposure at a hair salon with a universal face covering policy—Springfield, Missouri, May 2020. MMWR Morbidity and Mortality Weekly Report. 2020;69(28):930–932. doi: 10.15585/mmwr.mm6928e2 32673300

[7] Chu DK, Akl EA, Duda S, Solo K, Yaacoub S, Schünemann HJ, et al. Physical distancing, face masks, and eye protection to prevent person-to-person transmission of SARS-CoV-2 and COVID-19: A systematic review and meta-analysis. The Lancet. 2020;395(10242):1973–1987. doi: 10.1016/S0140-6736(20)31142-9 32497510 PMC7263814

[8] Leung NHL, Chu DKW, Shiu EYC, Chan KH, McDevitt JJ, Hau BJP, et al. Respiratory virus shedding in exhaled breath and efficacy of face masks. Nature Medicine. 2020;26(5):676–680. doi: 10.1038/s41591-020-0843-2 32371934 PMC8238571

[9] Betsch C. How behavioural science data helps mitigate the COVID-19 crisis. Nature Human Behaviour. 2020;4(5):438. doi: 10.1038/s41562-020-0866-1 32221514 PMC7101898

[10] Peeples L. Face masks: What the data say. Nature. 2020;586(7828):186–189. doi: 10.1038/d41586-020-02801-8 33024333

[11] Seres G, Balleyer A, Cerutti N, Friedrichsen J, Süer M. Face mask use and physical distancing before and after mandatory masking: Evidence from public waiting lines. Working Paper. 2020. 10.1016/j.jebo.2021.10.032 PMC8604556 34840368

[12] Seres Anna Helen Balleyer G, Cerutti N, Danilov Jana Friedrichsen DIW A, Berlin Yiming Liu HHU, Berlin Müge Süer WZB, Schmidt KM, et al. Face Masks Increase Compliance with Physical Distancing Recommendations during the COVID-19 Pandemic. Working Paper. 2020.

[13] Betsch C, Wieler LH, Habersaat K. Monitoring behavioural insights related to COVID-19. The Lancet. 2020;395(10232):1255–1256. doi: 10.1016/S0140-6736(20)30729-7 32247323 PMC7163179

[14] Omori R, Matsuyama R, Nakata Y. The age distribution of mortality from novel coronavirus disease (COVID-19) suggests no large difference of susceptibility by age. Scientific Reports. 2020;10(1):16642. doi: 10.1038/s41598-020-73777-8 33024235 PMC7538918

[15] Feng S, Shen C, Xia N, Song W, Fan M, Cowling BJ. Rational use of face masks in the COVID-19 pandemic. The Lancet Respiratory Medicine. 2020;8(5):434–436. doi: 10.1016/S2213-2600(20)30134-X 32203710 PMC7118603

[16] Greenhalgh T, Schmid MB, Czypionka T, Bassler D, Gruer L. Face masks for the public during the COVID-19 crisis. The BMJ. 2020;369. doi: 10.1136/bmj.m1435 32273267

[17] Tso RV, Cowling BJ. Importance of face masks for COVID-19: A call for effective public education. Clinical Infectious Diseases. 2020. doi: 10.1093/cid/ciaa593 PMC7337661 32614045

[18] Hoi G. Cover-up? How shifting policies affect Swiss attitudes toward masks; 2020. Available from: https://www.swissinfo.ch/eng/cover-up--how-shifting-policies-affect-swiss-attitudes-toward-masks/45978462.

[19] Stephens T. Should all Swiss be wearing face masks?; 2020. Available from: https://www.swissinfo.ch/eng/coronavirus_should-all-swiss-be-wearing-face-masks-/45663098.

[20] FOPH. Neues Coronavirus: Die Kampagne “So schützen wir uns” wechselt auf Pink; 2020. Available from: https://www.bag.admin.ch/bag/de/home/das-bag/aktuell/medienmitteilungen.msg-id-78968.html.

[21] WHO. Advice on the use of masks in the context of COVID-19; 2020. Available from: https://www.who.int/publications-.

[22] FOPH. New coronavirus; 2020. Available from: https://www.bag.admin.ch/bag/en/home/krankheiten/ausbrueche-epidemien-pandemien/aktuelle-ausbrueche-epidemien/novel-cov.html.

[23] Hale T, Angrist N, Cameron-Blake E, Hallas L, Kira B, Majumdar S, et al. Variation in government responses to COVID-19. Blavatnik School of Government Working Paper. 2020.

[24] Pfattheicher S, Nockur L, Böhm R, Sassenrath C, Petersen MB. The Emotional Path to Action: Empathy Promotes Physical Distancing and Wearing of Face Masks During the COVID-19 Pandemic. Psychological Science. 2020; p. 095679762096442. doi: 10.1177/0956797620964422 32993455

[25] Nakayachi K, Ozaki T, Shibata Y, Yokoi R. Why do Japanese people use masks against COVID-19, even though masks are unlikely to offer protection from infection? Frontiers in Psychology. 2020;11:1918. doi: 10.3389/fpsyg.2020.01918 32849127 PMC7417658

[26] Barceló J, Sheen GCH. Voluntary adoption of social welfare-enhancing behavior: Mask-wearing in Spain during the COVID-19 outbreak. PLOS ONE. 2020;15(12):e0242764. doi: 10.1371/journal.pone.0242764 33259531 PMC7707551

[27] Capraro V, Barcelo H. Telling people to “rely on their reasoning” increases intentions to wear a face covering to slow down COVID-19 transmission. Applied Cognitive Psychology. 2021; p. acp.3793. doi: 10.1002/acp.3793 33821089 PMC8013666

[28] Miguel FK, Machado GM, Pianowski G, Carvalho LdF. Compliance with containment measures to the COVID-19 pandemic over time: Do antisocial traits matter? Personality and Individual Differences. 2021;168:110346. doi: 10.1016/j.paid.2020.110346 32863507 PMC7441860

[29] Tobol Y, Siniver E, Yaniv G. Dishonesty and mandatory mask wearing in the COVID-19 pandemic. Economics Letters. 2020;197:109617. doi: 10.1016/j.econlet.2020.109617 33052153 PMC7543943

[30] Bhasin T, Butcher C, Gordon E, Hallward M, LeFebvre R. Does Karen wear a mask? The gendering of COVID-19 masking rhetoric. International Journal of Sociology and Social Policy. 2020;40(9-10):929–937. doi: 10.1108/IJSSP-07-2020-0293

[31] Blagov PS. Adaptive and Dark Personality in the COVID-19 Pandemic: Predicting Health-Behavior Endorsement and the Appeal of Public-Health Messages. Social Psychological and Personality Sci-ence. 2020; p. 194855062093643. doi: 10.1177/1948550620936439 PMC7342937 38602980

[32] Nowak B, Brzóska P, Piotrowski J, Sedikides C, Żemojtel-Piotrowska M, Jonason PK. Adaptive and maladaptive behavior during the COVID-19 pandemic: The roles of Dark Triad traits, collective narcissism, and health beliefs. Personality and Individual Differences. 2020;167:110232. doi: 10.1016/j.paid.2020.110232 32834282 PMC7363424

[33] Grant AM, Hofmann DA. It’s not all about me: Motivating hand hygiene among health care professionals by focusing on patients. Psychological Science. 2011;22(12):1494–1499. doi: 10.1177/0956797611419172 22075239

[34] Jordan J, Yoeli E, Rand DG. Don’t get it or don’t spread it? Comparing self-interested versus prosocial motivations for COVID-19 prevention behaviors. PsyArXiv. 2020; p. 1–58. 10.1038/s41598-021-97617-5 PMC8511002 34642341

[35] Betsch C, Böhm R, Korn L, Holtmann C. On the benefits of explaining herd immunity in vaccine advocacy. Nature Human Behaviour. 2017. doi: 10.1038/s41562-017-0056

[36] Kahneman D, Knetsch JL, Thaler RH. Fairness and the Assumptions of Economics. The Journal of Business. 1986. doi: 10.1086/296367

[37] McClintock CG. Social motivation—A set of propositions. Behavioral Science. 1972. doi: 10.1002/bs.3830170505

[38] Camerer CF, Fehr E. When does “economic man” dominate social behavior?; 2006. 10.1126/science.1110600 16400140

[39] Nowak MA, Sigmund K. Evolution of indirect reciprocity by image scoring. Nature. 1998. doi: 10.1038/31225 9634232

[40] Cheng KK, Lam TH, Leung CC. Wearing face masks in the community during the COVID-19 pandemic: Altruism and solidarity. The Lancet. 2020;0(0). doi: 10.1016/S0140-6736(20)30918-1 32305074 PMC7162638

[41] Oster E. Does disease cause vaccination? Disease outbreaks and vaccination response. Journal of Health Economics. 2018. doi: 10.1016/j.jhealeco.2017.10.003 29182938 PMC6522133

[42] Kantar Public. SOEP-Core 2018: Personenfragebogen, Stichproben A-L3 + N. SOEP Survey Papers. 2019;608:(Series A).

[43] Falk A, Becker A, Dohmen T, Enke B, Huffman D, Sunde U. Global evidence on economic preferences. Quarterly Journal of Economics. 2018;. doi: 10.1093/qje/qjy013

[44] Angrist JD, Pischke JS. Mostly Harmless Econometrics. Princeton: Princeton University Press; 2009.

[45] Hastie T, Tibshirani R, Wainwright M. Statistical learning with sparsity: The lasso and generalizations; 2015. doi: 10.1201/b18401

[46] Mullainathan S, Spiess J. Machine learning: An applied econometric approach. In: Journal of Economic Perspectives; 2017. doi: 10.1257/jep.31.2.87

[47] Arslan RC, Brümmer M, Dohmen T, Drewelies J, Hertwig R, Wagner GG. How people know their risk preference. Scientific Reports. 2020;10(1). doi: 10.1038/s41598-020-72077-5 32958788 PMC7505965

[48] Dohmen T, Falk A, Huffman D, Sunde U, Schupp J, Wagner GG. Individual risk attitudes: Measurement, determinants, and behavioral consequences. Journal of the European Economic Association. 2011. doi: 10.1111/j.1542-4774.2011.01015.x

[49] Seitz BM, Aktipis A, Buss DM, Alcock J, Bloom P, Gelfand M, et al. The pandemic exposes human nature: 10 evolutionary insights. Proceedings of the National Academy of Sciences. 2020; p. 202009787. doi: 10.1073/pnas.2009787117 33093198 PMC7668083

[50] Ariely D, Bracha A, Meier S. Doing good or doing well? Image motivation and monetary incentives in behaving prosocially. American Economic Review. 2009;99(1):544–555. doi: 10.1257/aer.99.1.544

[51] Andreoni J, Douglas Bernheim B. Social Image and the 50-50 Norm: A Theoretical and Experimental Analysis of Audience Effects. Econometrica. 2009;77(5):1607–1636. doi: 10.3982/ECTA7384

[52] Weber RA. Managing growth to achieve efficient coordination in large groups. American Economic Review. 2006;96(1):114–126. doi: 10.1257/000282806776157588

[53] Krupka EL, Weber RA. Identifying Social Norms Using Coordination Games: Why Does Dictator Game Sharing Vary? Journal of the European Economic Association. 2013;11(3):495–524. doi: 10.1111/jeea.12006

[54] Fong MW, Gao H, Wong JY, Xiao J, Shiu EYC, Ryu S, et al. Nonpharmaceutical measures for pandemic influenza in nonhealthcare settings-social distancing measures; 2020. 10.3201/eid2605.190995 PMC7181908 32027585

[55] Rose G. Sick individuals and sick populations. International Journal of Epidemiology. 2001;30(3):427–432. doi: 10.1093/ije/30.3.427 11416056

[56] Korn L, Böhm R, Meier NW, Betsch C. Vaccination as a social contract. Proceedings of the National Academy of Sciences of the United States of America. 2020;117(26):14890–14899. doi: 10.1073/pnas.1919666117 32541033 PMC7334515

[57] Bavel JJV, Baicker K, Boggio PS, Capraro V, Cichocka A, Cikara M, et al. Using social and behavioural science to support COVID-19 pandemic response; 2020. Available from: 10.1038/s41562-020-0884-z. 32355299

