# Supplementary material for: Wearing a mask—For yourself or for others? Behavioral correlates of mask wearing among COVID-19 frontline workers
Source: PLoS One. 2021 Jul 19;16(7):e0253621. doi: 10.1371/journal.pone.0253621 (PMC8289104; doi:10.1371/journal.pone.0253621)
Supplement: S1 Appendix — (PDF) [file pone.0253621.s001.pdf]

Wearing a mask—for yourself or for others? Behavioral correlates of mask wearing among COVID-19 frontline workers

Ankush Asri[1,2◑], Viola Asri[1,2◑], Baiba Renerte[1,2◑], Franziska Föllmi-Heusi[3,4], Joerg D. Leuppi[5,6], Juergen Muser[5], Reto Nüesch[3,6], Dominik Schuler[3,6], Urs Fischbacher[1,2],

1 University of Konstanz, Konstanz, Germany
2 Thurgau Institute of Economics, Kreuzlingen, Switzerland
3 Spital Schwyz, Schwyz, Switzerland
4 Swiss Institute for International Economics and Applied Economic Research, University of St.Gallen, Switzerland
5 Kantonsspital Baselland, Liestal, Switzerland
6 University of Basel, Basel, Switzerland

◑These authors contributed equally to this work.

* urs.fischbacher@uni-konstanz.de

Supplementary material

In this supplementary material, we present all survey questions, summary statistics and regression tables for the analyses related to the analysis in the paper.

1 Survey questions

We start the supplementary section by presenting all the variables we use in the analyses, the survey questions corresponding to these variables and how they are coded in the analyses. Table 1 below shows the details.

Table 1: Survey questions for all variables

**Survey question**	**Scale**	**Coded as**
**Dependent variable**		
Mask wearing		
Do you currently wear a mask when you go to supermarkets or other crowded places?	Yes<br>Sometimes<br>No	1<br>1<br>0
**Independent variables of interest**		
Risk aversion		
How do you see yourself? Are you in general a person willing to take risks or do you try to avoid risks?	Likert-scale from 0 to 10	Risk averse if < median<br>Risk loving otherwise

Altruism		
How well does this statement describe you as a person: “I am willing to donate to good causes without expecting anything in return.”	Likert-scale from 0 to 10	Altruistic if > median<br>Not altruistic otherwise
**Individual covariates**		
Age group		
Please enter your age in years:	Under 24 years<br>25-34 years<br>35-44 years<br>45-54 years<br>55-64 years<br>65 years or older	Older if<br>age > median age<br>Younger otherwise
Education group		
What is your highest level of education attained?	No education<br>or primary school<br>Realschule<br>(type of secondary<br>school)<br>Secondary school<br>High school<br>Apprenticeship<br>Vocational<br>Matura<br>Higher technical<br>school<br>University of<br>Applied Sciences<br>University	Dummy variables for<br>low, medium and high<br>education:<br>Low – up to secondary<br>school/A-level<br>Medium – Apprenticeship,<br>vocational training,<br>higher technical school<br>High – University of Applied<br>Sciences/University
Swiss native		
Were you born in Switzerland?	Yes<br>No	1<br>0
Gender		
Please indicate your gender:	Female<br>Male<br>No answer	1<br>2<br>3

Health workers		
What is your professional activity in the hospital?	Doctors<br>Nursing staff<br>Administration<br>Technical support<br>Other support	1<br>1<br>0<br>0<br>0
**Situational covariates**		
COVID-19 symptoms		
Have you had symptoms of flu or cold since July 2020?	Yes<br>No	1<br>0
Household size		
If you count yourself, how many people – children included – regularly live in your household?	Continuous	Continuous
Public transport		
How do you currently go to work?	On foot or bicycle<br>Car or Motorcycle<br>Public transport<br>Work from home	0<br>0<br>1<br>0
COVID-19 contact at work		
Were/are you in occupational contact with COVID-19 patients or their specimens (e.g., in A9, emergency department, laboratory)?	Yes<br>No<br>Do not know	1<br>0<br>0
COVID-19 contact outside work		
Have you been in close contact outside of work with a person who tested positive for COVID-19?	Yes<br>No<br>Do not know	1<br>0<br>0
COVID-19 risk group		
Do you count yourself in a COVID-19 risk group?	Yes<br>No<br>Do not know<br>Not specified	1<br>0<br>0<br>0

COVID-19 risk group household member		
Do you live in a household with someone who is in a COVID-19 risk group?	Yes<br>No<br>Do not know<br>Not specified	1<br>0<br>0<br>0
International travel		
Have you travelled abroad for at least 2 days since July 1, 2020?	Yes<br>No	1<br>0

2 Summary statistics

Table 2 shows the summary statistics for all variables included in the regression analysis separately for the more-affected and the less-affected regions.

Table 2: Summary statistics

	More-affected region				Less-affected region			
	mean	sd	min	max	mean	sd	min	max
Wearing a mask	0.609	0.49	0	1	0.193	0.40	0	1
Risk averse	0.478	0.50	0	1	0.447	0.50	0	1
Altruistic	0.378	0.49	0	1	0.330	0.47	0	1
Health worker	0.581	0.49	0	1	0.670	0.47	0	1
<24 years	0.081	0.27	0	1	0.090	0.29	0	1
25-34 years	0.252	0.43	0	1	0.257	0.44	0	1
35-44 years	0.257	0.44	0	1	0.240	0.43	0	1
45-54 years	0.219	0.41	0	1	0.217	0.41	0	1
>=55 years	0.191	0.39	0	1	0.197	0.40	0	1
Low education	0.094	0.29	0	1	0.093	0.29	0	1
Medium education	0.620	0.49	0	1	0.707	0.46	0	1
High education	0.285	0.45	0	1	0.200	0.40	0	1
Born in CH	0.665	0.47	0	1	0.780	0.41	0	1
Female	0.772	0.42	0	1	0.843	0.36	0	1
Male	0.228	0.42	0	1	0.150	0.36	0	1
Not identified	0.000	0.00	0	0	0.007	0.08	0	1
Had symptoms	0.478	0.50	0	1	0.533	0.50	0	1
Household size	2.857	1.27	1	8	2.993	1.49	1	13
Public transport	0.256	0.44	0	1	0.087	0.28	0	1
COVID-19 contact at work	0.620	0.49	0	1	0.423	0.49	0	1
COVID-19 contact outside	0.065	0.25	0	1	0.057	0.23	0	1
COVID19 risk group member	0.113	0.32	0	1	0.090	0.29	0	1
Household member COVID-19 risk group	0.215	0.41	0	1	0.190	0.39	0	1
International travel	0.378	0.49	0	1	0.197	0.40	0	1
Observations	540				300			

3 Linear regressions

We run linear probability regressions for the two samples from the two regions separately. The first specification does not include covariates. The second specification includes individual covariates, the third specification includes situational covariates, and the fourth specification includes individual and situational covariates.

Table 3: Linear regression - more-affected region

	(1)	(2)	(3)	(4)
Risk averse	0.135***	0.131***	0.128***	0.119***
	(0.0415)	(0.0413)	(0.0414)	(0.0416)
Altruistic	0.0959**	0.0681	0.0944**	0.0656
	(0.0424)	(0.0422)	(0.0422)	(0.0424)
Adj. R-Squared	0.024	0.074	0.048	0.080
Individual covariates	No	Yes	No	Yes
Situational covariates	No	No	Yes	Yes
Observations	540	540	540	540

Dependent variable is mask wearing in shops and other public places. Individual covariates include age, education, occupation, native and gender. Situational covariates include having had symptoms, household size international travel, using public transport, having been in contact with COVID-19 at work or outside work, and having a household member who belongs to COVID-19 risk group. Standard errors in parentheses.
* $p < 0.1$, ** $p < 0.05$, *** $p < 0.01$

Table 4: Linear regression - less-affected region

	(1)	(2)	(3)	(4)
Risk averse	0.117**	0.108**	0.107**	0.0981**
	(0.0467)	(0.0459)	(0.0459)	(0.0461)
Altruistic	0.0799	0.0325	0.0701	0.0327
	(0.0509)	(0.0504)	(0.0502)	(0.0490)
Adj. R-Squared	0.026	0.115	0.063	0.131
Individual covariates	No	Yes	No	Yes
Situational covariates	No	No	Yes	Yes
Observations	300	300	300	300

Dependent variable is mask wearing in shops and other public places. Individual covariates include age, education, occupation, native and gender. Situational covariates include having had symptoms, household size international travel, using public transport, having been in contact with COVID-19 at work or outside work, and having a household member who belongs to COVID-19 risk group. Standard errors in parentheses.
* $p < 0.1$, ** $p < 0.05$, *** $p < 0.01$

We include interaction terms between *older* and the variables of interest *risk averse* and *altruistic*.

Table 5: Linear regression - risk aversion and altruism with age

	(1)<br>More-affected region	(2)<br>Less-affected region
Risk averse	0.115**	0.110**
	(0.0538)	(0.0550)
Older	0.182***	0.142*
	(0.0690)	(0.0753)
Risk averse X Older	0.0135	-0.0443
	(0.0813)	(0.0994)
Altruistic	0.125**	0.0328
	(0.0574)	(0.0562)
Altruistic X Older	-0.130	0.00656
	(0.0853)	(0.1025)
Adj. R-Squared	0.084	0.114
Individual covariates	Yes	Yes
Situational covariates	Yes	Yes
Observations	540	300

Dependent variable is mask wearing in shops and other public places. Individual covariates include age, education, occupation, native and gender. Situational covariates include having had symptoms, household size international travel, using public transport, having been in contact with COVID-19 at work or outside work, and having a household member who belongs to COVID-19 risk group. Standard errors in parentheses.

* $p < 0.1$, ** $p < 0.05$, *** $p < 0.01$

4 Linear regressions with continuous independent variables of interest

Below, we present our regression analysis using continuous independent variables of interest for risk preference and pure altruism. The variables are standardized with mean value 0 and standard deviation of 1. The first specification does not include covariates. The second specification includes individual covariates, the third specification includes situational covariates, and the fourth specification includes individual and situational covariates.

Table 6: Linear regression - more-affected region

	(1)	(2)	(3)	(4)
Risk averse	0.0878***	0.0797***	0.0842***	0.0746***
	(0.0199)	(0.0204)	(0.0204)	(0.0208)
Altruistic	0.0366*	0.0233	0.0372*	0.0239
	(0.0208)	(0.0208)	(0.0203)	(0.0204)
Adj. R-Squared	0.035	0.080	0.058	0.086
Individual covariates	No	Yes	No	Yes
Situational covariates	No	No	Yes	Yes
Observations	540	540	540	540

Dependent variable is mask wearing in shops and other public places. Individual covariates include age, education, occupation, native and gender. Situational covariates include having had symptoms, household size international travel, using public transport, having been in contact with COVID-19 at work or outside work, and having a household member who belongs to COVID-19 risk group. Standard errors in parentheses.
* $p < 0.1$, ** $p < 0.05$, *** $p < 0.01$

Table 7: Linear regression - less-affected region

	(1)	(2)	(3)	(4)
Risk averse	0.0516**	0.0519**	0.0453*	0.0460*
	(0.0243)	(0.0242)	(0.0234)	(0.0241)
Altruistic	0.0323	0.0117	0.0242	0.00819
	(0.0220)	(0.0216)	(0.0229)	(0.0221)
Adj. R-Squared	0.018	0.112	0.054	0.127
Individual covariates	No	Yes	No	Yes
Situational covariates	No	No	Yes	Yes
Observations	300	300	300	300

Dependent variable is mask wearing in shops and other public places. Individual covariates include age, education, occupation, native and gender. Situational covariates include having had symptoms, household size international travel, using public transport, having been in contact with COVID-19 at work or outside work, and having a household member who belongs to COVID-19 risk group. Standard errors in parentheses.
* $p < 0.1$, ** $p < 0.05$, *** $p < 0.01$

We include interaction terms between *older* and the variables of interest *risk averse* and *altruistic*.

Table 8: Linear regression - risk aversion and altruism with age

	(1) More-affected region	(2) Less-affected region
Risk averse	0.0783***	0.0478*
	(0.0282)	(0.0278)
Older	0.131***	0.121**
	(0.0426)	(0.0571)
Older X Risk averse	-0.00484	-0.0156
	(0.0399)	(0.0494)
Altruistic	0.0421*	0.0188
	(0.0255)	(0.0257)
Older X Altruistic	-0.0455	-0.0160
	(0.0417)	(0.0467)
Adj. R-Squared	0.089	0.110
Individual covariates	Yes	Yes
Situational covariates	Yes	Yes
Observations	540	300

Dependent variable is mask wearing in shops and other public places. Individual covariates include age, education, occupation, native and gender. Situational covariates include having had symptoms, household size international travel, using public transport, having been in contact with COVID-19 at work or outside work, and having a household member who belongs to COVID-19 risk group. Standard errors in parentheses.
* $p < 0.1$, ** $p < 0.05$, *** $p < 0.01$

5 Probit regressions

Below, we present probit regressions given the binary dependent variable. The first specification does not include covariates. The second specification includes individual covariates, the third specification includes situational covariates, and the fourth specification includes individual and situational covariates.

Table 9: Marginal effects from probit regression - more-affected region

	(1)	(2)	(3)	(4)
Risk averse	0.136^{***}	0.136^{***}	0.134^{***}	0.127^{***}
	(0.0425)	(0.0439)	(0.0434)	(0.0446)
Altruistic	0.0981^{**}	0.0690	0.0980^{**}	0.0673
	(0.0440)	(0.0453)	(0.0448)	(0.0458)
Individual covariates	No	Yes	No	Yes
Situational covariates	No	No	Yes	Yes
Observations	540	540	540	540

Dependent variable is mask wearing in shops and other public places. Individual covariates include age, education, occupation, native and gender. Situational covariates include having had symptoms, household size international travel, using public transport, having been in contact with COVID-19 at work or outside work, and having a household member who belongs to COVID-19 risk group. Standard errors in parentheses.
* $p < 0.1$, ** $p < 0.05$, *** $p < 0.01$

Table 10: Marginal effects from probit regression - less-affected region

	(1)	(2)	(3)	(4)
Risk averse	0.116^{***}	0.111^{**}	0.106^{**}	0.103^{**}
	(0.0450)	(0.0437)	(0.0441)	(0.0432)
Altruistic	0.0774^{*}	0.0404	0.0748	0.0376
	(0.0468)	(0.0454)	(0.0461)	(0.0436)
Individual covariates	No	Yes	No	Yes
Situational covariates	No	No	Yes	Yes
Observations	300	298	300	298

Dependent variable is mask wearing in shops and other public places. Individual covariates include age, education, occupation, native and gender. Situational covariates include having had symptoms, household size international travel, using public transport, having been in contact with COVID-19 at work or outside work, and having a household member who belongs to COVID-19 risk group. Standard errors in parentheses.
* $p < 0.1$, ** $p < 0.05$, *** $p < 0.01$

We include interaction terms between *older* and the variables of interest: *risk aversion* and *altruism.*

Table 11: Marginal effects from probit regression - risk aversion and altruism with age

	(1) More-affected region	(2) Less-affected region
Risk averse	0.126^{**}	0.116^{**}
	(0.0566)	(0.0536)
Older	0.144^{***}	0.129^{**}
	(0.0448)	(0.0531)
Older X Risk averse	0.124^{**}	0.0656
	(0.0630)	(0.0807)
Altruistic	0.131^{**}	0.0406
	(0.0605)	(0.0518)
Older X Altruistic	-0.0111	0.0340
	(0.0646)	(0.0857)
Individual covariates	Yes	Yes
Situational covariates	Yes	Yes
Observations	540	298

Dependent variable is mask wearing in shops and other public places. Individual covariates include age, education, occupation, native and gender. Situational covariates include having had symptoms, household size international travel, using public transport, having been in contact with COVID-19 at work or outside work, and having a household member who belongs to COVID-19 risk group. Standard errors in parentheses.
* $p < 0.1$, ** $p < 0.05$, *** $p < 0.01$

6 Ordered Probit regressions

Below, we present ordered probit regressions given the continuous dependent variable. The first specification shows the marginal effects for the respondents not wearing a mask, the second specification shows the marginal effects for the respondents wearing a mask only sometimes, and the third specification shows marginal effects for the respondents wearing a mask all the time. All specifications includes individual and situational covariates.

Table 12: Marginal effects from ordered probit regression - more-affected region

	Mask wearing		
	(1)	(2)	(3)
	No	Sometimes	Yes
Risk averse	-0.0852**	0.0171*	0.0681**
	(0.0386)	(0.0091)	(0.0305)
Altruistic	-0.0357	0.00718	0.0285
	(0.0390)	(0.0081)	(0.0311)
Individual covariates	Yes	Yes	Yes
Situational covariates	Yes	Yes	Yes
Observations	540	540	540

Dependent variable is mask wearing in shops and other public places. Individual covariates include age, education, occupation, native and gender. Situational covariates include having had symptoms, household size international travel, using public transport, having been in contact with COVID-19 at work or outside work, and having a household member who belongs to COVID-19 risk group. Standard errors in parentheses.

* $p < 0.1$, ** $p < 0.05$, *** $p < 0.01$

Table 13: Marginal effects from ordered probit regression - less-affected region

	Mask wearing		
	(1)	(2)	(3)
	No	Sometimes	Yes
Risk averse	-0.118***	0.103***	0.0143*
	(0.0433)	(0.0382)	(0.0079)
Altruistic	-0.0243	0.0214	0.00296
	(0.0425)	(0.0376)	(0.0050)
Individual covariates	Yes	Yes	Yes
Situational covariates	Yes	Yes	Yes
Observations	300	300	300

Dependent variable is mask wearing in shops and other public places. Individual covariates include age, education, occupation, native and gender. Situational covariates include having had symptoms, household size international travel, using public transport, having been in contact with COVID-19 at work or outside work, and having a household member who belongs to COVID-19 risk group. Standard errors in parentheses.

* $p < 0.1$, ** $p < 0.05$, *** $p < 0.01$

We include interaction terms between *older* and the variables of interest: *risk aversion* and *altruism.*

Table 14: Marginal effects from ordered probit regression - risk aversion and altruism with age

	More-affected region			Less-affected region		
	(1) No	(2) Sometimes	(3) Yes	(4) No	(5) Sometimes	(6) Yes
Risk averse	-0.0856*	0.0253	0.0603*	-0.129**	0.115**	0.0142
	(0.0516)	(0.0163)	(0.0363)	(0.0555)	(0.0479)	(0.0098)
Older	-0.121***	0.0202**	0.101***	-0.138**	0.118**	0.0200*
	(0.0392)	(0.0082)	(0.0335)	(0.0544)	(0.0461)	(0.0114)
Older X Risk averse	-0.0822	0.000470	0.0818	-0.0864	0.0692	0.0172
	(0.0525)	(0.0067)	(0.0521)	(0.0790)	(0.0639)	(0.0165)
Altruistic	-0.0870	0.0242*	0.0628	-0.0229	0.0206	0.00231
	(0.0538)	(0.0146)	(0.0404)	(0.0493)	(0.0445)	(0.0050)
Older X Altruistic	0.0164	-0.000284	-0.0161	-0.0248	0.0199	0.00488
	(0.0532)	(0.0017)	(0.0521)	(0.0825)	(0.0662)	(0.0164)
Individual covariates	Yes	Yes	Yes	Yes	Yes	Yes
Situational covariates	Yes	Yes	Yes	Yes	Yes	Yes
Observations	540	540	540	300	300	300

Dependent variable is mask wearing in shops and other public places. Individual covariates include age, education, occupation, native and gender. Situational covariates include having had symptoms, household size international travel, using public transport, having been in contact with COVID-19 at work or outside work, and having a household member who belongs to COVID-19 risk group. Standard errors in parentheses.

* $p < 0.1$, ** $p < 0.05$, *** $p < 0.01$

7 Lasso regressions

To address the concern of having arbitrarily selected covariates in the previously presented regression models, we present here a data-driven approach for the selection of covariates.

Table 15: Lasso covariate-selection

	(1)	(2)
Risk averse	0.135***	0.109**
	(0.0405)	(0.0478)
Altruistic	0.0897**	0.0460
	(0.0414)	(0.0519)
P-value variables of interest	0.000	0.040
Individual covariates	Yes	Yes
Situational covariates	Yes	Yes
Observations	540	300

Dependent variable is mask wearing in shops and other public places. Individual covariates include age, education, occupation, native and gender. Situational covariates include having had symptoms, household size international travel, using public transport, having been in contact with COVID-19 at work or outside work, and having a household member who belongs to COVID-19 risk group. Standard errors in parentheses.
* $p < 0.1$, ** $p < 0.05$, *** $p < 0.01$

Table 16: Lasso covariate-selection - risk aversion and altruism with age

	(1) More-affected region	(2) Less-affected region
Risk averse	0.123**	0.115**
	(0.0534)	(0.0543)
Older	0.174***	0.161**
	(0.0667)	(0.0632)
Risk averse X Older	0.0134	-0.0243
	(0.0804)	(0.0969)
Altruistic	0.136**	0.0546
	(0.0562)	(0.0575)
Altruistic X Older	-0.148*	0.0204
	(0.0830)	(0.0987)
P-value variables of interest	0.000	0.000
Individual covariates	Yes	Yes
Situational covariates	Yes	Yes
Observations	540	300

Dependent variable is mask wearing in shops and other public places. Individual covariates include age, education, occupation, native and gender. Situational covariates include having had symptoms, household size international travel, using public transport, having been in contact with COVID-19 at work or outside work, and having a household member who belongs to COVID-19 risk group. Standard errors in parentheses.
* $p < 0.1$, ** $p < 0.05$, *** $p < 0.01$
